# Endoscopic suture-based closure of an enterocutaneous fistula after one-anastomosis gastric bypass

**DOI:** 10.1055/a-2503-5984

**Published:** 2025-01-14

**Authors:** Patrick Rebnegger, Antonia Gantschnigg, Michael Grechenig, Klaus Emmanuel, Josef Holzinger, Franz Singhartinger

**Affiliations:** 1 31545 Department of Surgery, Paracelsus Medical University Salzburg, Salzburg, Austria

Gastrointestinal cutaneous fistulas are complex and challenging surgical conditions that occur predominantly postoperatively 1 . Effective treatment requires a multidisciplinary approach, with surgical revision carefully planned and typically considered after the failure of conservative treatments 2 . Endoscopic therapies, including clipping and vacuum therapy, along with newly developed endoscopic suture-based techniques, have shown promising results in closing fistulas without the high morbidity and mortality of surgical interventions 3 4 5 .

We present the case of a 64-year-old man who developed an enterocutaneous fistula following a laparoscopic one-anastomosis gastric bypass for morbid obesity. His postoperative course was complicated by perforation at the biliopancreatic limb. After two unsuccessful attempts at permanent closure surgically and a subsequent subcutaneous infection, an enterocutaneous fistula developed. A temporary fistula closure was achieved using an over-the-scope (OTS) clip but, after displacement of the clip ( Fig. 1 **a** ), the fistula persisted.

**Fig. 1:**
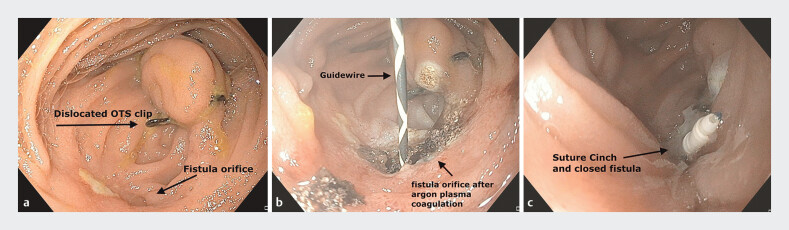
Endoscopic views showing: **a** the displaced over-the-scope (OTS) clip and the fistula orifice; **b** the fistula orifice after argon plasma coagulation (marked with a guidewire); **c** the suture cinch and closed fistula.

Endoscopic fistula closure was performed 10 months postoperatively. This was carried out using an endoscopic helix tacking system (X-Tack; Boston Scientific Corporation, Marlborough, Massachusetts, USA) after argon plasma coagulation of the fistula tract had been performed ( Fig. 1 **b,c** ; Video 1 ), and was followed by placement of an external suture to close the cutaneous opening of the fistula. The patient’s postinterventional course was uneventful. At follow-up after 2 months, no recurrence of the fistula was detected.

Endoscopic closure of an enterocutaneous fistula using an endoscopic helix tacking system after argon plasma coagulation of the fistula tract.Video 1

Endoscopic enterocutaneous fistula closure is safe and feasible after failed surgery. Novel endoscopic suturing devices extend the possibilities for a tailored approach regarding different locations and types of fistula.

Endoscopy_UCTN_Code_TTT_1AO_2AZ

## Notes

Endoscopy E-Videos https://eref.thieme.de/e-videos
*E-Videos* is an open access online section of the journal *Endoscopy* , reporting on interesting cases and new techniques in gastroenterological endoscopy. All papers include a high-quality video and are published with a Creative Commons CC-BY license. Endoscopy E-Videos qualify for HINARI discounts and waivers and eligibility is automatically checked during the submission process. We grant 100% waivers to articles whose corresponding authors are based in Group A countries and 50% waivers to those who are based in Group B countries as classified by Research4Life (see: https://www.research4life.org/access/eligibility/ ). This section has its own submission website at https://mc.manuscriptcentral.com/e-videos .
